# Pulmonary dysfunction in overweight and obese children with obstructive sleep apnoea

**DOI:** 10.1002/edm2.417

**Published:** 2023-04-06

**Authors:** Aina Salwa Kasim, Shahram Golbabapour, Azriyanti Anuar Zaini, Eg Kah Peng, Muhammad Yazid Jalaludin, Anna Marie Nathan, Karuthan Chinna, Surendran Thavagnanam

**Affiliations:** ^1^ Department of Paediatrics University of Malaya Kuala Lumpur Malaysia; ^2^ Rheumatology Research Group, Institute of Inflammation and Ageing University of Birmingham, Queen Elizabeth Hospital Birmingham UK; ^3^ University Malaya Paediatric and Child Health Research Group University of Malaya Kuala Lumpur Malaysia; ^4^ UCSI university Kuala Lumpur Kuala Lumpur Malaysia

**Keywords:** children, cytokines, obesity, obstructive sleep apnoea, overweight, pulmonary function

## Abstract

**Introduction:**

Overweight and obese children are at risk of obstructive sleep apnoea (OSA) and abnormal pulmonary function (PF).

**Aim:**

Investigate the relationship between body mass index (BMI), OSA on PF in children.

**Materials & Method:**

Seventy‐four children were recruited. Mixed obstructive apnoea‐hypopnea index (MOAHI), BMI, oxygen saturation (SpO_2_), forced expiratory volume one second (FEV_1_), forced vital capacity (FVC) and fractionated exhaled nitric oxide (FeNO) were measured.

**Results:**

Twenty‐four and thirty children had mild OSA and moderate‐to‐severe OSA respectively. BMI correlated negatively with SpO_2_ nadir (*r* = −.363, *p* = .001). FVC, FEV_1_ and nadir SpO_2_ values decreased with OSA severity (*p <* .001). The odds of a child with OSA having an abnormal spirometry was 3.16 (95% CI: 1.08, 9.22). There was significant association between FeNO and AHI (*r* = .497, <.001).

**Discussion:**

Overweight and obese children with OSA have significant abnormalities in pulmonary function independent of BMI. OSA severity and elevated FeNO also correlated with diminishing lung function.

## Graphical abstract

Childhood obesity is on the rise and is associated with an increased risk of sleep disordered breathing, metabolic complications and later adult obesity. This is the first paper to demonstrate a relationship between abnormal lung function, obesity and obstructive sleep apnoea.

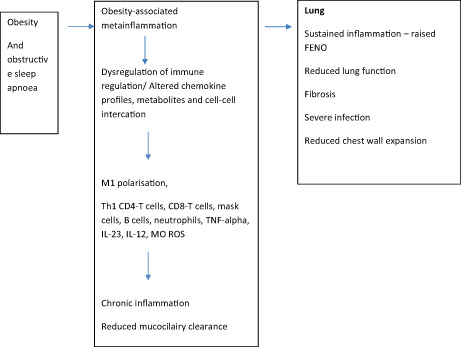

## 1 INTRODUCTION

With the emerging obesity epidemic, up to 60% of overweight and obese (OO) children are diagnosed with obstructive sleep apnoea (OSA)^1^, ^2^ and both conditions may independently or synergistically influence system inflammation.^3^ It has been proposed that the adult sleep apnoea syndrome is related to sleep apnoea in children, and that the differences of OSA in patient populations of different age groups may represent different stages in the development of the adult form of OSA.^4^

Several studies have reported pulmonary dysfunction (PD) in adults with OSA^5^, ^6^; however, limited data is available regarding the association among OO, OSA and PD in children. Van Eyck et al. found a correlation between OSA severity and diminished lung function, reporting that obesity being an important confounding factor in both OSA severity and pulmonary dysfunction.^7^ Obesity has been recognized as a risk factor for the development of asthma with altered pulmonary function, poor treatment response and high morbidity.^8^, ^9^ The incidence of asthma is 1.47 times higher in obese individuals than in non‐obese individuals, and a three‐unit increase in BMI is associated with a 35% increase in the risk of asthma.^10^ In a cohort study of more than 25,000 children and adults with asthma, Schatz et al.^11^ showed that a higher BMI was associated with worsened asthma control and an increased risk of asthma exacerbations.

Pathophysiologically, OSA may be on the causal pathway between asthma and obesity. Plausible mechanisms by which sleep apnoea may exacerbate asthma are through enhanced vagal cholinergic tone induced by apnoea, upper airway repetitive obstruction‐induced local airway inflammation, systemic inflammation and apnoea‐related alterations in intrathoracic pressures, the latter resulting in bronchoconstriction. It is also plausible that low‐grade inflammation and oxidative stress generated during apnoea‐related hypoxaemia episodes could attribute to airway inflammation and bronchial hyperactivity.^3^ Carpagnano et al. found that fractionated exhaled nitric oxide (FeNO) was elevated in OSA patient with obesity and positively correlated with obstructive sleep apnoea severity^12^; however, limited data is known about the presence of FeNO in children with OSA.

Although the relationship between obesity and pulmonary dysfunction is becoming increasingly clear, there is still much controversy regarding whether it occurs in OO children with OSA. Therefore, the primary objective of this study was to investigate the relationship between OSA and PFT in OO children. We also sought to investigate if the metabolic inflammation would drive airway inflammation leading to airway hyperactivity in obese children with and without asthma.

## 2 MATERIALS AND METHODS

In this study, consecutive OO children (aged 6–13 years) with suspected OSA were recruited from the paediatric clinic and sleep laboratory at University Malaya Medical Centre (UMMC) between March 2016 and May 2017. In this study, children with BMI 25 kg/m^2^ were classified as overweight and 30 kg/m^2^ as obese as defined by the International Obesity Task Force criteria.^13^ Children who were unable to perform spirometry; on supplemental oxygen or non‐invasive ventilation; with chronic lung disease; neuromuscular or craniofacial syndromes were excluded. None of the patients had adenotonsillectomy at the time of this study. Informed consent was obtained from the parents/legal guardians and the ethics committee of UMMC approved this study (MECID 20162–2139). All patients recruited were given a validated questionnaire to assess their symptoms of sleep‐disordered breathing (SDB).^14^ All measured variables including body measurement, spirometry, polysomnography, fractionated exhaled nitric oxide, and bloods for cytokines, eosinophils and neutrophils were done within 3 months of each other with no significant changes in body measurement and SDB symptoms during this interval.

### 2.1 Demographic data

Sociodemographic information was collected from both parents and patients. The diagnosis of asthma was based on history, clinical examination and investigations.

### 2.2 Pulmonary function testing

All participants underwent standard spirometry in line with American Thoracic Society/European Respiratory Society guidelines. Lung function (FEV1, FVC, FEF 25%–75%) was performed in sitting position using the Vmax Encore Spirometer (CareFusion Respiratory).The best spirometric measure of at least three reproducible attempts was recorded for analysis. Reference values from Morris/Polgar were used with ethnic corrections.^15^

### 2.3 Polysomnography

All polysomnography (PSG) studies were conducted according to the American Academy of Sleep Medicine (AASM) manual for the scoring of sleep by sleep machine (Cadwell Kennewick/Compumedics)^16^ and manually scored by paediatric sleep specialists. Recorded respiratory data included counts and indices of the following events: obstructive apnoeas, obstructive hypopneas, central apnoeas and mixed apnoeas during sleep. The OSA severity was defined as the number of obstructive apnoeas, hypopneas and mixed apnoeas per hour (MOAHI). Children were scored according to the paediatric OSA severity scoring criteria: mild OSA = MOAHI ≥ 1.5 – <5/h; moderate OSA = MOAHI ≥ 5 – <10/h; and severe OSA = MOAHI ≥10/h.

### 2.4 Fractionated exhaled nitric oxide

Exhaled nitric oxide was measured according to American Thoracic Society/European Respiratory Society guidelines recommendations^17^ using portable NIOX MINO analyser (Aerocrine). While seated comfortably, the patients FeNO was measured using the standardized online single—breath technique with exhalation at a constant flow rate of 50 mL/s, until a nitric oxide plateau of at least 2 s can be identified during an exhalation of at least 4 s. Patients were refrained from eating and drinking 1 h prior to FeNO measurement. FeNO measurements were also deferred in patients with respiratory tract infection until they had recovered. FeNO levels in parts per billion (ppb) were considered normal at <25 ppb as per ATS guidelines.

### 2.5 Quantitative measurements of inflammatory cytokines; blood neutrophils and eosinophils

Blood was drawn for full blood count to look at percentage of neutrophil and eosinophil counts, and the expression level of inflammatory cytokines in plasma using a human Inflammation immune‐assays kit (Bio‐Rad Laboratories) as per manufacturer's instructions. The cytokine analysis was performed by MAGPIX, a Luminex® Instrument (R&D Systems). A standard curve was derived using the different concentrations of the assay standard. Intra—assay variability was represented as the coefficient of variation as per manufacturer's instructions. Internal control was used for data normalization.

### 2.6 Statistical analysis

Statistical analysis was performed using IBM SPSS statistics version 23.0 (IBM Corp). Categorical variables summarized as frequencies and percentages. Normally distributed continuous variables were presented as means and standard deviations while skewed variables were described as medians and interquartile ranges. For comparing group differences, a multivariate analysis was performed and the ANOVA procedure was used for normally distributed data and the nonparametric Kruskal–Wallis test was used for non‐normal data. When significant differences were observed, post hoc tests were used to test pairwise difference. In testing the associations between continuously variables, either Pearson's or Spearman's correlations were used. For all tests, the significant values were set at *p <* .05.

## 3 RESULTS

### 3.1 Characteristics of participants

Between 1st March 2016 and 31st May 2017, 303 children were seen at the paediatric clinic and sleep laboratory. Out of this, 229 were excluded from the study 148 did not fulfil the study criteria and 81 had incomplete data (either no polysomnography or spirometry data). In the final analysis, there were 74 children. In this study, majority (70.3%) of the patients were Malays and the mean age was 10.71 ± SD 2.1 years. 32 (43.2%) of the patients had asthma. Only one patient had adenotonsillar hypertrophy. The median BMI was 28.7 (IQR 7.16) kg/m2, mean z score was 2.37 ± SD 0.44 and median percentage body fat mass was 26.7 (IQR 11.4). Of the 74 patients, 20 (27.1%) had no OSA, 24 (32.4%) had mild OSA and 30 (40.5%) had moderate–to‐severe OSA. There were no significant associations between OSA groups and patient characteristics (Table 1). With an increase in BMI, there was a significant decrease with the SpO2 nadir (*p* = −.363, *p* = .001). There was no significant difference in baseline SpO_2_ between the OSA groups (*p* = .052), but nadir SpO_2_, decreased significantly with OSA severity (*p* < .001).

**TABLE 1:** Patient characteristics, nocturnal respiratory parameters and awake pulmonary functions by oAHI groups.

	oAHI ≤ 1	1.5 > oAHI ≤ 5	oAHI ≥ 5	*p*‐value
	*n* = 20	*n* = 24	*n* = 30	
Male/Female	17/3	21/3	19/11	.072
Age (years) (Min, Max)	10.3 ± 2.3 (5, 13)	8.9 ± 2.8 (4, 13)	8.7 ± 2.6 (5, 13)	.054
Height (cm)	146.2 ± 16.5	147.3 ± 18.2	143.8 ± 18.2	.763
Weight (kg)	62.3 ± 19.5	63.6 ± 23.1	69.9 ± 29.7	.521
BMI z‐score	2.26 ± 0.45	2.33 ± 0.47	2.46 ± 0.41	.265
BFM (%)	40.1 ± 10.8	41.3 ± 9.3	44.5 ± 9.2	.542
Asthma (%)	7 (35%)	14 (58%)	11 (37%)	.191
Nocturnal respiratory parameters				
Baseline SpO_2_	99 (2)	98 (3)	98 (3)	.052
Nadir SpO_2_ ^*^	93 (4)	90 (8)	84 (13)	<.001
Awake pulmonary function				
FVC^*^	91.8 ± 15.1	82.2 ± 11.7	76.4 ± 11.4	<.001
FEV_1_ ^*^	92.6 ± 16.0	83.3 ± 12.3	74.9 ± 14.5	<.001
FEV_1_/FVC	90.0 ± 7.2	87.7 ± 8.7	85.8 ± 7.5	.193
FEF_25%–75%_	95.9 ± 30.1	84.5 ± 30.0	80.5 ± 28.8	.182

Abbreviations: BFM, body fat mass; BMI, body mass index; FEF, forced expiratory flow 25%–75%; FEV1, forced expiratory volume at 1 s; FVC, forced vital capacity; Mean ± SD; Median (IQR); oAHI, obstructive apnoea‐hypopnoea index.

* Significant result, *p* < .05.

Overall, 41 (55.5%) of the children had abnormal spirometry results where 14 (18.9%) had obstructive spirometry and 27 (36.5%) had restrictive pattern. About 63% of OSAS children had abnormal spirometry of which 23 (68%) had restrictive pattern (*χ* ^*2*^ (2) = 6.89, *p* = .032). The odds of a child with OSA having an abnormal spirometry was 3.16 (95% CI: 1.08, 9.22). Among the awake pulmonary functions, mean FVC and FEV_1_ decreased significantly with increasing OSA severity (*p* < .001), but not for FEV_1_/FVC and FEF_25%–75%_. There was no significant association between OSA severity and the presence of abnormal spirometry result (*χ* ^*2*^ (2) = 4.99, *p =* .08).

Children with moderate to severe OSA had significantly lower mean FVC (*p* < .001) and FEV_1_ (*p* < .001) values compared to the children with no or mild OSA (Table 1). There were moderate correlations between AHI score, nadir SpO_2_ and two spirometry parameters. AHI score negatively correlated with FVC (*r* = −.385, *p* < .001) and FEV1 (*r* = −.409, *p* < .004), while nadir SpO_2_ positively correlated with FEV_1_ (*r* = .275, *p* = .018) (Table 2). In univariate analyses, FEV_1_, FVC, FEF_25–75_ and BMI were significantly associated with OSA severity. In multivariate analysis, only FEV_1_ (*p* < .001) and BMI (Mod/Sev OSA, *p* = .017 and Mild OSA, *p* = .013) were significant (Table 3). There were no significant differences in AHI values, SpO_2_ nadir, FEV_1_, FVC, FEV_1_/FVC and FEF_25%–75%_ between those with asthma and without asthma (Table 4). There was also no significant correlation between measures of adiposity including BMI z‐score and percentage of body fat mass with any spirometry parameters (Table 5).

**TABLE 2:** Association between pulmonary function tests and polysomnography parameters.

	AHI (Spearman's *r*; *p*)	Nadir SpO2 (Spearman's *r; p*)
FVC	−.385; .001*	.190; .105
FEV_1_	−.409; <.001*	.275; .018*
FEV_1_/FVC	−.193; .100	.142; .226
FEF_25%–75%_	−.210; .073	.132; .263

Abbreviations: FEF, forced expiratory flow; FEV1, forced expiratory volume at 1 s; FVC, forced vital capacity.

* Significant result, *p* < .05.

**TABLE 3:** Multivariate analysis between spirometry, BMI and OSA severity.

Variable	OSA severity	OR (95% CI)	*p*‐value
FEV1	Mod/Sev	0.913 (0.868, 0.962)	.001
	Mild	0.965 (0.926, 1.007)	.102
	Normal	1	
BMI	Mod/Sev	3.154 (1.227, 8.109)	.017
	Mild	2.145 (1.172, 3.928)	.013
	Normal	1	

**TABLE 4:** Association between spirometry and polysomnography parameters and asthma.

	Asthma present (*n* = 32)	Asthma absent (*n* = 43)	*t*‐value	*p*‐value
SpO_2_ nadir	86.81 ± 6.87	86.29 ± 8.96	0.276	.783
FEV_1_	85.84 ± 14.02	79.81 ± 16.69	1.648	.104
FVC	83.25 ± 14.03	81.83 ± 13.94	0.432	.667
FEV_1_/FVC	87.84 ± 7.68	87.31 ± 8.19	0.286	.776
FEF_25%–75%_	82.88 ± 28.23	88.50 ± 31.08	0.802	.425
AHI values	4.15 (IQR, 7.60)	2.30 (IQR, 6.20)	0.276	.785

Abbreviations: FEF, forced expiratory flow; FEV1, forced expiratory volume at 1 s; FVC, forced vital capacity; Mean ± SD, Median ± IQR.

**TABLE 5:** Correlation analysis between spirometry parameters and BMI z‐score and % body fat mass.

	BMI z‐score (Correlation *r*; *p*‐value)	% body fat mass (Correlation *r*; *p*‐value)
FVC	.075; *p* .524	−.174; p .288
FEV1	−.016; *p* .892	−.126; *p* .446
FEV1/FVC	−.017; *p* .883	−.132; *p* .422
FEF_25‐75%_	.211; *p* .071	−.037; *p* .825

Abbreviations: BMI, body mass index; FEF, forced expiratory flow; FEV1, forced expiratory volume at 1 s; FVC, forced vital capacity.

### 3.2 Fractioned exhaled nitric oxide (FeNO)

Out of the 74 children, 46 (62%) had their FeNO measured, of which 11 (15%) children had no OSA, 16 (22%) had mild OSA and 19 (26%) had moderate to severe OSA. The median FeNO value was 23.0 (IQR 3.0) parts per billion. The median FeNO value in the moderate and severe OSA group was significantly higher compared to the normal group (*p* = .019). FeNO negatively correlated with FEV_1_ (*r* = −.366, *p* = .012), FVC (*r* = −.487, *p* = .001), FEF_25–75%_ (*r* = −.307, *p* = .038) and positively correlated with AHI (*r* = .497, *p* < .001) (Table 6). However, FeNO did not significantly correlate with BMI (*r* = .011, *p* = .942). FeNO levels measured were also not statistically significant between asthmatics and non‐asthmatics in this study (z‐value = −.033, *p* = .974).

**TABLE 6:** Associations between spirometry parameters, AHI and FeNO levels.

Parameters	FeNO (Spearman's *r*; *p*‐value)
FEV1	−.366, .012*
FVC	−.487, .001*
FEV1/FVC	−.159, .292
FEF_25%–75%_	−.307, .038*
AHI	.497, <.001*

Abbreviations: FEF, forced expiratory flow; FEV1, forced expiratory volume at 1 s; FVC, forced vital capacity.

* Significant result, *p* < .05.

### 3.3 Inflammatory cytokines and blood neutrophils and eosinophils measurements

Plasma samples from 30 (40.5%) children (9 children without OSA, 12 with mild OSA and 9 with moderate—severe OSA) were assessed for the level of inflammatory cytokines. There were no significant associations between, both BMI and OSA, and cytokines levels (Table 7). Serum neutrophil and eosinophil counts were sampled from 48 children as a measure of systemic inflammation. Median eosinophils count (%) was 1.95 (IQR 4.66) and mean neutrophil count (%) was 52.77 ± 11.69. There was no significant association between either % eosinophils or % neutrophils count with OSA or BMI. There was no significant difference in % mean neutrophils or eosinophils count between asthmatics (52.85 ± 5.05) and non‐asthmatics (*p* = .967 and *p* = .714) respectively.

**TABLE 7:** Association between AHI, BMI Z– score, and cytokines levels.

Cytokines	BMI z score (Pearson's *r*; *p*‐ value)	AHI (Spearman's *r*; *p*‐value)
IL—2	.14; .943	.251; .181
IL—4	−.091; .632	−.004; .983
IL—5	.113; .553	−.050; .794
IL—6	.208, .27	.203; .283
IL—8	−.027; .887	.153; .421
IL—9	−.065; .733	.307; .099
IL—13	.237; .207	.135; .477
TNF—α	−.097; .611	−.070; .711

Abbreviations: BMI, mody mass index; IL, interleukin; TNF, tumour necrosis factor.

## 4 DISCUSSION

This study investigated the relationship between lung function and OSA severity in children with high BMI. The major finding from this study is that OO children with OSA have significant abnormalities in pulmonary function compared to those without OSA especially FEV_1_ and FVC. FeNO correlated significantly with OSA severity (positively) and lung function (negatively) in OO children with OSA irrespective of asthma diagnosis.

The frequency of OSA in our study population was higher than previously proposed.^2^ The possible explanation for the higher prevalence of OSA in our patient cohort could be due to known distinguished changes to the upper airway structure and facial geometry in Asian children.^18^ With the emerging obesity epidemic, along with an increased risk of OSA due to the craniofacial structure in children of Asian descent, local paediatricians should be vigilant in screening for OSA in children.

Almost 56% of the children in this study had an abnormal spirometry with restrictive pattern being twice as much as obstructive pattern.^19^ Of these group of children, 80% had OSA. Pulmonary function correlated with OSA severity; increased OSA severity correlated with diminished lung function, especially FEV_1_ and FVC. Desaturation during sleep was associated with worse FEV_1_. Obesity and sleep apnoea both may cause changes in thoracic mechanics resulting in dysfunction of lung compliance and reduced lung capacity.^19^, ^20^ The FEV_1_/FVC ratio is generally well preserved or elevated even in morbidly obese individuals, indicating that FEV_1_ and FVC are affected at the same rate.^21^ By reducing functional lung volume, obesity can change airway diameter due to the interdependence of the airway and the adjacent pulmonary parenchyma which may explain the changes seen in our patients' spirometry results. Because of the ineffectiveness of the respiratory muscles, there is closure of dependent airways with the formation of small areas of atelectasis and increased alveolar surface tension due to a reduction in FRC.^22^ All of these factors lead to inspiratory overload which increases respiratory effort, heterogeneity of ventilation distribution and respiratory energy expenditure and therefore, increases neural respiratory drive, in addition to causing respiratory sleep disorders and eventually hypercapnic respiratory failure.^23^ A study by Van Eyck et al.^19^ demonstrated both restrictive and obstructive spirometry patterns, with FEV_1_ similarly diminished with worsening OSA severity without significant corresponding reduction of FVC; however, they had a smaller population diagnosed with OSA (18.3%).

In our study, there was significant correlation between FeNO levels with OSA severity^24^ and spirometry parameters, specifically FEV_1_, FVC and FEF_25%–75%_ independent of BMI. One possible reason for the observed higher levels of FeNO in our study could be due to ethnic differences. FeNO levels are signifiable higher in Asian children due to their genetic regulation of nitric oxide synthase pathway.^25^, ^26^ Meta‐analysis performed by Zhang D et al. showed FeNO was significantly increased in subjects with OSA especially on waking up. This was likely due to upper airway inflammation from mucosal injury secondary to intermittent airway closure and reopening as well as ischemia–reperfusion injury from intermittent nocturnal hypoxemia. In another experiment by Verhulst et al.^27^ involving the paediatric population, habitual snoring and age were the only variables associated with raised FENO levels in the morning and afternoon, leading them to conclude that snoring is more important than the actual obstructive respiratory events for increased upper airway inflammation. Local upper airway inflammation then promotes oropharyngeal inspiratory muscle dysfunction and the formation of progressive local neurogenic lesions, thus amplifying the upper airway narrowing and collapsibility.^28^ This may then explain the pulmonary dysfunction findings in our study. Long term CPAP therapy has been shown to reduced inflammation‐related infiltration in upper airways.^28^ Thus, FENO can be used as a simple, non‐invasive marker of upper airway inflammation in patients with OSA, allowing a more accurate prediction of response to treatment.

There was also no significant difference between obese children with asthma and without asthma with regards to lung function and OSA severity. Inflammatory cytokines in plasma and the percentage of neutrophils and eosinophils did not show significant correlation with OSA and obesity. This suggests that there are probably independent local and systemic inflammatory pathways involved in obesity and OSA. Other possible explanations for this could be due to the relative younger age of the patients in our study cohort, with shorter duration of OSA and without relatively severe OSA. The study does not find a relationship between local inflammatory cytokines collected by FeNO and plasma from peripheral blood but cytokine analysis on sputum or bronchoalveolar lavage may help understand the exact relationship between local inflammation, OSA, OO and pulmonary dysfunction.

This study has considerable strengths, including a population of younger children of different ethnicity to investigate possible mechanism for pulmonary dysfunction in obese children with OSA. The procedures were performed uniformly by trained personnel for measuring lung function, FeNO and performing sleep studies in children. On the other hand, several limitations should be acknowledged. Firstly, this is a single centre study with small sample size. The patients were recruited from sleep laboratory and paediatric clinic therefore resulting in a higher prevalence of OSA. However, this does not influence the main aim of the study looking at the association between OO children and OSA on pulmonary function. Secondly, some of the spirometry and polysomnography may have been performed as much as 3 months apart due to appointment issues; however, as there was minimal change in the BMI or OSA symptoms, if there are any discrepancies, it is likely to be negligible. Thirdly, we were only able to perform FeNO measurements on 62% patients due to lack of funding, but we were able to demonstrate significant relationship between FeNO, OSA and pulmonary dysfunction, which warrants further investigations. Fourthly, it would have been good to have obtained bronchoalveolar lavage samples for cytokine analysis to study mechanistic pathways associated between OSA, obesity and pulmonary dysfunction. Although the association between asthma and obesity remained uncertain until recently, the existence of different asthma phenotypes is now well recognized^29^ which could explain the negative findings in our study.

## 5 CONCLUSION

Abnormalities between awake pulmonary function and sleep respiratory parameters may be observed in Malaysian population of children with OSA independent of a higher BMI and/or asthma diagnosis. Diminished spirometry is associated with OSA severity. The correlation between FEV_1_, FeNO and OSA severity implies a possible role for local inflammation in the pathology of OSA.

## AUTHOR CONTRIBUTIONS

**Aina Salwa Kasim:** Data curation (equal); formal analysis (equal). **Shahram Golbabapour:** Investigation (equal); methodology (equal). **Eg Kah Peng:** Writing – review and editing (equal). **Anna Marie Nathan:** Funding acquisition (supporting); methodology (supporting); writing – review and editing (equal). **Muhammad Yazid Jalaludin:** Funding acquisition (supporting); writing – review and editing (equal). **Azriyanti Anuar Zaini:** Conceptualization (supporting); investigation (supporting); project administration (supporting); supervision (equal); writing – review and editing (supporting). **Karuthan Chinna:** Formal analysis (lead); methodology (supporting); validation (equal). **surendran thavagnanam:** Conceptualization (lead); formal analysis (equal); funding acquisition (lead); methodology (lead); project administration (lead); supervision (lead); visualization (lead); writing – original draft (lead).

## CONFLICT OF INTEREST STATEMENT

The author has no conflict of interest to declare.

## Data Availability

The data that support the findings of this study are available from the corresponding author upon reasonable request.
